# Comparative evaluation of real-time PCR and ELISA for the detection of human fascioliasis

**DOI:** 10.1038/s41598-024-54602-y

**Published:** 2024-02-16

**Authors:** Fatemeh Bakhshipour, Mohammad Zibaei, Mohammad Bagher Rokni, Abolfazl Miahipour, Farzaneh Firoozeh, Masoud Beheshti, Leila Beikzadeh, Gita Alizadeh, Mojgan Aryaeipour, Vahid Raissi

**Affiliations:** 1 Department of Parasitology and Mycology, School of Medicine, Alborz University of Medical Sciences, P.O. Box: 3149779453, Karaj, Iran; 2 Department of Parasitology and Mycology, School of Public Health, Tehran University of Medical Sciences, Tehran, Iran; 3 Center for Research of Endemic Parasites of Iran, Tehran University of Medical Sciences, Tehran, Iran; 4 Department of Microbiology, School of Medicine, Alborz University of Medical Sciences, Karaj, Iran; 5 Department of Virology, School of Medicine, Ahvaz Jundishapur University of Medical Sciences, Ahvaz, Iran; 6 Department of Medical Laboratory Sciences, Faculty of Para-Medicine, Alborz University of Medical Sciences, Karaj, Iran; 7 Department of Parasitology and Mycology, School of Medicine, Iran University of Medical Sciences, Tehran, Iran

**Keywords:** *Fasciola*, Serum, Human, Real-time PCR, ELISA, Immunology, Microbiology, Molecular biology

## Abstract

Fascioliasis is a zoonotic parasitic infection caused by *Fasciola* species in humans and animals. Despite significant advances in vaccination and new therapeutic agents, little attention has been paid to validating methods for the diagnosis of fascioliasis in humans. Serological techniques are convenient assays that significantly improves the diagnosis of *Fasciola* infection. However, a more sensitive method is required. The aim of this study was to compare the Real-Time PCR technique with the indirect-ELISA for the detection of *Fasciola hepatica* in human. Using a panel of sera from patients infected with *Fasciola hepatica* (*n* = 51), other parasitic infections (*n* = 7), and uninfected controls (*n* = 12), we optimized an ELISA which employs an excretory–secretory antigens from *F. hepatica* for the detection of human fascioliasis. After DNA extraction from the samples, molecular analysis was done using Real-Time PCR technique based on the *Fasciola* ribosomal ITS1 sequence. Of 70 patient serum samples, 44 (62.86%) samples were identified as positive *F. hepatica* infection using ELISA and Real-Time PCR assays. There was no cross-reaction with other parasitic diseases such as toxoplasmosis, leishmaniasis, taeniasis, hydatidosis, trichinosis, toxocariasis, and strongyloidiasis. The significant difference between the agreement and similarity of the results of patients with indirect ELISA and Real-Time PCR was 94.4% and 99.2%, respectively (Cohen’s kappa ≥ 0.7; *P* = 0.02). Based on the Kappa agreement findings, the significant agreement between the results of ELISA and Real-Time PCR indicates the accuracy and reliability of these tests in the diagnosis of *F. hepatica* in humans.

## Introduction

Fascioliasis, a zoonoses helminthic infection with a worldwide distribution, causing substantial economic losses in the animal husbandry industry, including anthelmintic treatments, control of intermediate hosts (molluscicides), and the implication of economic losses in dairy and meat livestock production^1^. *Fasciola hepatica* and *Fasciola gigantica* are trematode parasites responsible for fascioliasis which is also increasingly recognized as a disease in humans and animals^2^. Human infection with *Fasciola* species has existed from prehistoric times to the present and has a significant impact on global health in specific geographic locations^3^. Fascioliasis, an important helminthic zoonosis, is classified by World Health Organization (WHO) as a neglected tropical disease with an estimated 17 million people infected and about 180 million people living in endemic areas at risk to infection^3,4^. The prevalence of fascioliasis infections is related to people’s eating habits. Aquatic plants consumed collected from wetlands are an important factor in transmission to humans^5^.

Human infection is commonly recognized based on such symptoms as fever, headache, epigastric pain, right upper abdominal pain, malaise, and nausea. Based on clinical manifestation time, fascioliasis is classified as acute (0–4 months) and chronic (more than12 months)^6^. The early diagnosis of *Fasciola* infection is critical for the prevention of chronic infection with possible mortality^7^.

Human fascioliasis is generally detected by conventional parasitological tests to recover eggs in the stool, serological tests for determination of anti-*Fasciola* antibodies, and molecular approaches to detect *Fasciola* DNA^8–10^. Although faecal examination and microscopic observations of worm eggs is known as the gold standard for the identification of *Fasciola*, it is time-consuming and unsuccessful for acute fascioliasis^11^. This method has disadvantages, including: infected people do not start ovulating until they have been infected for several months; patients in the acute stage of infection do not excrete eggs. Therefore, in the early stages, the infection should be diagnosed by other methods than examination of feces in the laboratory^12^. The serological tests are cost-effective, rapid, and high sensitive; nonetheless, the detection of antibodies does not always indicate active fascioliasis, and people from endemic areas generally show weak serological responses. Accordingly, in the endemic areas for *Fasciola* species in human, the diagnostic titer of sera depends on levels of endemicity^13^. Among the serological tests, the Enzyme-Linked Immunosorbent Assay (ELISA) is the most commonly used method for the detection of fascioliasis^14^. ELISA represents a sensitive and pragmatic means of detecting fascioliasis, with positivity typically manifesting two weeks following infection^15^. Nonetheless, there has limitations to using the mentioned serological test for the detection of incomplete/blocking antibodies and as well as its long-term positivity for several months after treatment in chronic patients in chronic patients^15,16^. In such cases, molecular-based methods are very sensitive techniques that can be used due to the fact that they allow the rapid amplification of a specific part of DNA^17^.

The use of diagnostic methods based on Polymerase Chain Reaction (PCR) has steadily increased in the field of parasitology in recent decades due to low cost, high sensitivity, and specificity. According to previous reports, PCR is reliable for the early diagnosis and detection of acute or chronic fascioliasis^18,19^. Quantitative PCR (qPCR), also called Real-Time PCR, is a well-established method for the detection, quantification, and typing of different parasitic agents in the areas of clinical and veterinary diagnostics^20^.

The purpose of this study was to evaluate the Real-Time PCR method for the detection of *Fasciola* DNA in serum and to compare their sensitivity and specificity using human samples tested with indirect-ELISA as a comparison.

## Materials and methods

### Ethics approval and consent to participate

The research ethical approval for the study was granted by the Ethical Committee at the University of Medical Sciences in Alborz, and informed consent obtained from the participants prior to data collection (IR.ABZUMS.REC.1401.032). Written informed consent form was received from each study participant. All methods were carried out in accordance with relevant guidelines and regulations.

### Patients and sera

Seventy serum samples were used for this study. Fifty-one out of 70 sera studied were from people with hepatobiliary fascioliasis. All patients were underwent upper abdominal sonography and Computed Tomography (CT) scanning. The human *Fasciola* samples were reviewed and approved by the “National Reference Laboratory for Diagnosis of Fasciolosis” School of Health, Tehran University of Medical Sciences (TUMS, Tehran, Iran). The human control and other parasitic diseases sera were obtained from the Helminthology Research Laboratory of the Department of Parasitology and Mycology, Alborz University of Medical Sciences, Iran (ABZUMS, Karaj, Iran). All samples used in this study were anonymized. The samples were assigned to five groups as follows:

*Group A*: 34 positive and suspected fascioliasis serology samples. *Group B*: 10 samples from patients whose fascioliasis test was positive by ELISA method and *Fasciola* eggs were observed in their feces (considered definitively positive cases). *Group C*: 12 control samples were taken from healthy individuals who showed negative serology and did not have detectable anti-*Fasciola* antibodies. *Group D*: 7 serum samples were obtained from individuals infected with other parasitic diseases such as toxocariasis, strongyloidiasis, taeniasis, hydatidosis, trichinosis, toxoplasmosis, and leishmaniasis. *Group E*: 7 ELISA-positive samples that were treated and referred for a second evaluation, so that molecular studies were performed to further investigate the presence of fascioliasis.

### Preparation of *Fasciola* excretory/secretory antigens

*Fasciola hepatica* Excretory/Secretory Antigens (ESAgs) were prepared from adult worms as previously described with some modifications^21^. In brief, *F. hepatica* adult worms were collected from the livers of infected cattle and washed 3–4 times at room temperature with Phosphate Buffered Saline (PBS) (0.01 M, pH 7.2) for 1 h at 37 °C. The adult worms were cultivated in RPMI 1640 (Sigma-Aldrich Chemie GmbH, Germany) medium supplemented with sodium bicarbonate (Merck, Darmstadt, Germany; 8.5% μg/mL), L-glutamine (20 mM), 4-(2-hydroxyethyl) piperazine-1-ethanesulfonic acid (HEPES) (Merck), penicillin/streptomycin (100 IU, 100 μg/mL), and 1% glucose (Merck, Darmstadt, Germany). Culture supernatant was exchanged daily and pooled; then dialyzed overnight against 5 mM acetate buffer (pH 5) at 4 °C, protease inhibitors (phenylmethylsulfonyl fluoride) were added, concentrated, and stored at − 70 °C. The protein content was measured by the Bradford protein assay^22^.

### Enzyme-linked immunosorbent assay

ELISA microplates (Nunc, Roskilde, Denmark) were coated with 1 mg/mL of *F. hepatica* ESAgs (100 μL/well) in coating buffer (0.05 M carbonate–bicarbonate buffer, pH 9.6) and incubated overnight at 37 °C. Plates were washed five times in phosphate-buffered saline containing 0.05% Tween 20 (PBST). After removing unbound coating antigen, the excess binding sites were blocked with 3% skimmed milk in PBST and plates incubated for 2 h at 37 °C. The wells were washed in PBST and 100 μL of diluted (1:500) serum samples were added to the plates and incubated at 37 °C for 30 min. The plates were washed again and 100 μL of diluted anti-human IgG horseradish-peroxidase conjugated (Sigma-Poole, UK) at a 12,000-fold dilution in PBST (1:12,000) was added to each well and incubated for 30 min at 37 °C. After washing three times, the plates were incubated with chromogen/substrate [100 μg/well of o-phenylenediamine dihydrochloride (OPD)], 0.025% H2O2 in 0.1 M citrate buffer pH 5) and were stopped by addition of 50 μL of 1 M sulfuric acid after 30 min. The optical density (OD) of samples was monitored at a wave length of 492 nm using a microplate reader. The cut-off point was set as the mean optical density of the negative controls plus two standard deviations.

### Serum DNA extraction

DNA extraction was performed for all reference and samples using the genomic DNA kit (DNG™-PLUS), according to the manufacturer’s instructions with some modifications^23^.

### Primers

The primers used in PCR and Real-Time PCR techniques targeting ribosomal Internal Transcribed Spacer 1 (ITS1) and Glyceraldehyde-3-phosphate dehydrogenase (GAPDH) region of *F. hepatica* genome were adapted from a previous study^23^. The primers were synthesized by Sinaclon Company (Tehran, Iran) and deposited in the GenBank. The sequences of the primers used in this study are listed in Table 1.

**Table 1:** Primers used for PCR and Real-Time PCR and sequencing.

Primers	Sequences	References
ITS1	Forward: TGGTATGCTTGCGTCTCTCG	^23^
	Reverse: GCCGTAGCCCAAATCTCCTC	
GAPDH	Forward: CCCACTCCTCCACCTTTGAC	
	Reverse: TTTTCTGAGCCAGCCACCAG	

### PCR and real-time PCR

PCR was conducted in a final volume of 25 μL containing 12.5 μL of master mix (Ampliqon), 1 μL of the primers, 2 μL of extracted DNA and 9.5 μL of distilled water. The detailed PCR temperature cycling conditions as follows: one cycle of primary initial denaturation at 95 °C for 5 min, followed by 35 cycles of denaturation at 95 °C for 30 s, annealing at 55 °C for 30 s, and elongation at 72 °C for 1 min. The final cycle was followed by extension at 72 °C for 5 min. The PCR products were separated by electrophoresis on a 1.5% agarose gel and visualized with a UV transilluminator (UV Transilluminator, QUANTUM SD4-1000, VILBER, France) after staining with 3 μg/mL GelRed. Real-time PCR was performed using species-specific primers and probes for detecting *F.* hepatica^23^. Ready-to-use Real-Time PCR master mix (6.5 μL) (QuantiTect Probe PCR master mix; Qiagen, Hilden, Germany) was used with 2 μL of each primer, 3.5 μL of distilled water, and 3 μL of DNA. The thermal cycling conditions in Real-Time PCR system were 95 °C for 5 min (Initial denaturation), 40 cycles of 95 °C for 30 s (Denaturation), 55 °C for 30 s (Annealing), and 72 °C for 1 min (Extension), and by a melting curve stage of 95 °C for 10 s and 65 °C for 60 s.

### Statistical analysis

The Kappa agreement test was used to determine the agreement between the methods. Statistical analysis was done using SPSS software version 26 (IBM Corp., Armonk, NY, USA). Chi-square (χ2) and Fisher’s exact tests were used to evaluate associations of the variables. *P*-value less than 0.05 was considered statistically significant.

## Result

### Optimization of ELISA using ESAgs for diagnosis of fascioliasis

Out of 70 serum samples collected from the study subjects, 44 samples (62.86%) were found to be infected with fascioliasis using ESAgs of *F. hepatica* and ELISA that including 34 patients who were previously diagnosed as seropositive for fascioliasis (Group A) and 10 people who were seropositive and *Fasciola* eggs recovered from fecal samples using parasitology assay and stool examination (Group B).

### PCR assay

**Figure 1:**
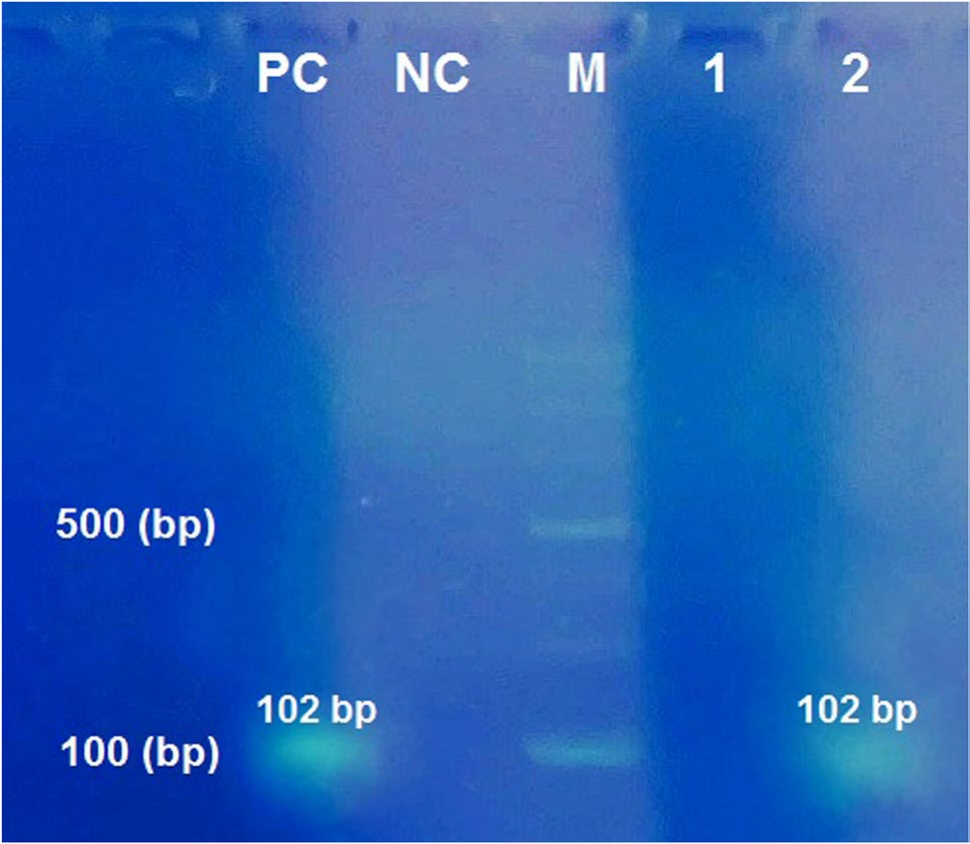
Analysis of PCR products amplified of *Fasciola* species from serum samples by 1.5% agarose gel electrophoresis. *PC* Positive control with genomic DNA, *NC* Negative control without DNA, *M* Molecular size marker 100-bp, Lane 1: negative sample, Lane 2: *Fasciola hepatica* with ITS1/GAPH primers (a band of 102-bp was observed). Full-length gel image is presented in Supplementary Fig. 1.

Electrophoresis of PCR products on 1.5% agarose gel stained with DNA safe stain showed that among 70 DNA samples extracted from serum samples, 44 specimens were diagnosed positive with *F. hepatica* and analysis of PCR products revealed positive band (102-bp) in fascioliasis patients (Fig. 1).

### Agreement between real-time PCR and indirect ELISA

In the current study, the housekeeping gene (HKG) was detected in the samples, which increases the accuracy of the positive or negative results obtained. There was a high similarity in the results of the patients (more than 99%) and only about 5% difference in the results with the two methods (Cohen’s kappa coefficient: 0.71; *P* = 0.02). Accordingly, the similarity of patients’ results and agreement (based on Kappa statistics) were found to be 99.2% and 94.4%, respectively (Table 2).

**Table 2:** Agreement and similarity of indirect ELISA and real-time PCR results.

Samples, N (%)	Real-time PCR	ELISA		*P*-value	Agreement (%)	Coefficient Cohen’s kappa
		Positive, N (%)	Negative, N (%)			
Untreated						
Group A, 34 (54%)	Negative	0 (0.0)	0 (0.0)	0.01^†^	100.0	1.00
	Positive	34 (100.0)	0 (0.0)			
Group B, 10 (15.9%)	Negative	0 (0.0)	0 (0.0)	0.01	100.0	1.00
	Positive	10 (100.0)	0 (0.0)			
Group C, 12 (19%)	Negative	0 (0.0)	11 (91.7)	0.03	80.7	0.88
	Positive	0 (0.0)	1 (8.3%)			
Group D, 7 (11.1%)	Negative	0 (0.0)	7 (100.0)	0.01	100.0	1.00
	Positive	0 (0.0)	0 (0.0)			
Total, 63 (100%)	Negative	0 (0.0)	18 (28.5)	0.02	94.4	0.71
	Positive	44 (69.9)	1 (1.6)			
Treated						
Group E 7 (10%)	Negative	7 (100)	0 (0.0)	0.97	0.0	0.01
	Positive	0 (0.0)	0 (0.0)			
Total, 70 (100%)	Negative	7 (10.0)	18 (25.7)	0.02	82.3	0.95

^†^Significance by the *χ*^2^ test.

**Figure 2:**
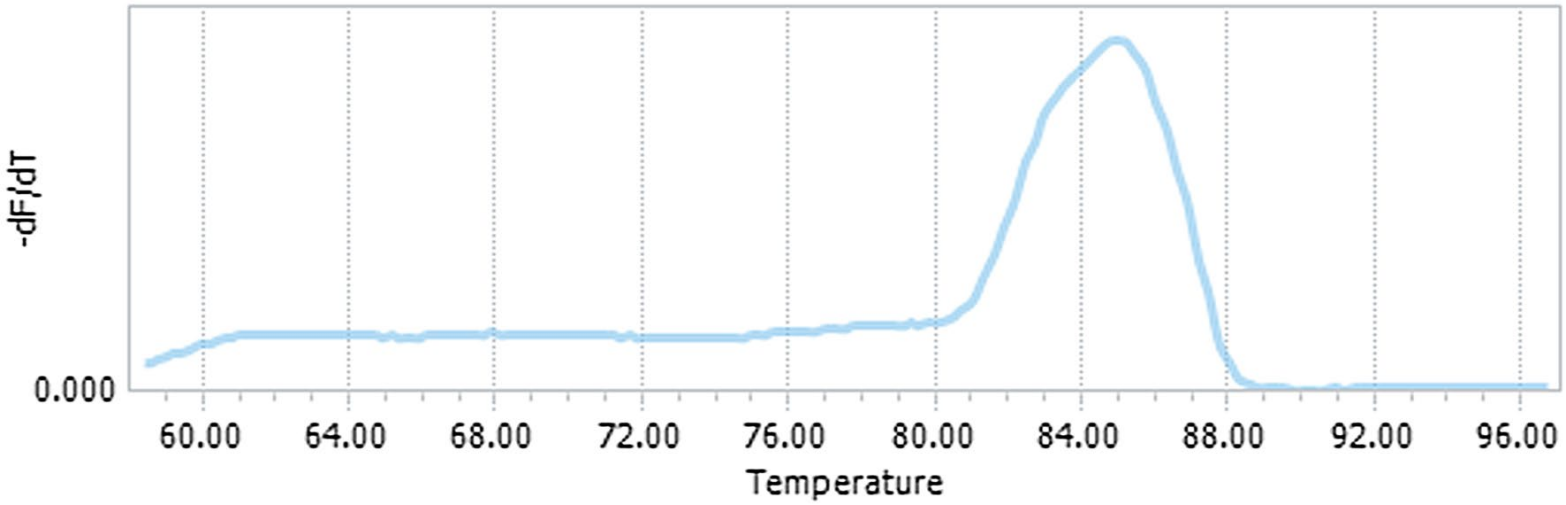
A melting curve analysis of ITS-1 real-time PCR showing species-specific melting peaks for *Fasciola hepatica*.

Of the 34 ELISA positive samples suspected of fascioliasis (Group A), after molecular analysis, all positive serum samples using FES-ELISA were also Real-Time PCR positive, which showed that there is a significant level of agreement between the two diagnostic methods (*P* = 0.01) (Fig. 2). In the group that included 10 positive cases of fascioliasis and *Fasciola* eggs were observed in direct stool examination (Group B), people obtained positive results with both serological and Real-Time PCR methods, and a significant difference was observed between them. Eleven of 12 samples (Group C), the findings were negative for both molecular and serological methods. Group D included seven samples from patients with other parasitic diseases such as toxocariasis, strongyloidiasis, taeniasis, hydatidosis, and trichinosis, as well as toxoplasmosis and leishmaniasis. All these patients were negative for *Fasciola* infection by serological and molecular-based method, indicating the absence of cross-reactivity. Both the ELISA and Real-Time PCR yielded consistent results which indicates a high level of agreement between the two methods (*P* = 0.01). Finally, a group included seven samples that were referred for investigate after treatment (Group E). The ELISA findings were positive and the Real-Time PCR assay results were negative for fascioliasis. The results demonstrate a mismatch between these two approaches.

## Discussion

Although the clinical and economic importance of fascioliasis has been known for centuries, the available diagnostic tests are not well suited to detect infection in humans^24^.

In the current study, conventional serological test (indirect-ELISA), PCR and Real-Time PCR techniques were compared together for the diagnosis of *F. hepatica* in serum samples of human. Based on the acquired results, the most positive cases for *F. hepatica* were diagnosed by Real-Time PCR assays.

The intricate pathophysiology of *Fasciola* infection in humans may pose challenges for disease diagnosis and management, and gold standard approaches and/or experimental settings have mainly been used to evaluate diagnostic testing and their limitations^6,24–28^. While various diagnostic techniques are employed to diagnose human fascioliasis, each has its limitations and drawbacks. For instance, serological diagnosis may yield false-positive results due to cross-reactivity, also one of the disadvantages of serological methods is that positive tests remain more than six months after the treatment of the disease. Moreover, immune-deficient patients may not be accurately diagnosed using serological methods^8,29, 30^. In past studies, ELISA has been shown to be the most common method for diagnosing helminth infections, but serodiagnostic tests for parasitic diseases still pose problems, including cross-reactivity between them. Previous studies demonstrated that the sensitivity of ELISA for toxocariasis is 80% and its specificity is 90–95%. On the other hand, the findings shows that there may be common antigens and cross-reactivity between *Toxocara canis* and *Fasciola*, *Trichinella*, *Strongyloides* and *Schistosoma* species^31,32^. In a study by Cicek et al., cross-reactivity between whole worm extracts of *Fasciola* and *Toxocara* species was demonstrated, which it may be due to the effectiveness of the home-made ELISA method^33^. Parasitic helminths express various antigenic carbohydrates, which often account for serological cross-reactions. In previous investigate, it was found that there are common antigens between hydatidosis and fascioliasis and there is cross-positivity in the serological methods^34^. Although parasitological examinations are considered as the gold standard and the way of differential diagnosis in some cases, they are not useful for the acute diagnostic stages of human fascioliasis^29^. The majority of microscopy techniques exhibit low sensitivity and necessitate repeated testing and egg concentration. Furthermore, the sensitivity is even further diminished when egg counts are low, which can be observed in cases of long-term infections, treatment failure, or infections caused by hybrid parasites (*Fasciola* species)^10 ^Therefore, the molecular methods are more specific than the mentioned methods, their susceptibility to rapid contamination constitutes a limitation factor^35,36^. In modern parasitology, molecular methods based on DNA analysis have significant effects in many fields, including systematic determination of parasites, diagnosis of infections, analysis of parasite epidemics, investigation of genetic structures within parasite populations (such as genetic differences between genera), and research on gene expression, vaccine development, and drug resistance^37–39^.

Comparing diagnostic methods, including molecular, serological, and microscopic, has always been a subject of investigation in parasitic diseases to establish an initial and reference method. Since most studies on human fascioliasis have only focused on limited outlooks, evaluating the correlation between diagnostic and clinical aspects and response to treatment is essential.

The present study, which was designed to detect the agreement between indirect-ELISA and Real-Time PCR methods in the diagnosis of fasciolosis in endemic areas, the findings showed more than 94.4% agreement and 99.2% similarity. According to the results of Kappa statistics, our findings show that molecular methods can complement conventional direct stool examination, antigen and antibody-based methods for the detection, identification and epidemiologic analysis of *Fasciola* infection. This study was able to demonstrate significant differences between the agreement and similarity of results of indirect-ELISA and Real-Time PCR by Kappa index. In addition, all patients who were treated or seropositive were diagnosed as negative using Real-Time PCR method. Alizadeh et al., compared the Semi-Nested-PCR and ELISA for the diagnosis of *F. hepatica* in human serum^40^. Their results showed that Nested-PCR was a more sensitive method to detect *Fasciola* than serological assay. In another study, the performance of single-step duplex PCR was evaluated with stool samples^41^. Their results provide evidence to suggest that novel mitochondrial DNA (mtDNA) duplex PCR is a sensitive and fast tool for accurate identification of *Fasciola* species in areas of distributional and zonal overlap. One study reported 100% similarity between ELISA and Nested-PCR in the diagnosis of chronic toxoplasmosis^42^. Our study confirms these findings and demonstrates the sensitivity and value of molecular methods. In the study by Olaogun and colleagues, they compared three diagnostic methods—sedimentation technique, Copro-ELISA, and qPCR—for detecting *Fasciola* in cattle, with the molecular approach demonstrating the best sensitivity^43^. In a study conducted to identify strongyloidiasis in serum samples obtained from immunocompromised patients, it was demonstrated that the molecular method utilizing cell-free DNA present in human serum samples is indeed feasible^44^. In the present study, we detected fascioliasis in serum samples using a molecular technique targeting cell-free DNA. This approach effectively addressed the issue of false negative results associated with the serology method, which can arise due to the absence of antibodies in some patients. In other studies, researchers have proven that molecular-based methods for distinguishing between *Fasciola hepatica* and *Fasciola gigantica* are particularly useful for epidemiological research^45,46^. The present study confirms previous results and provides further evidence that molecular techniques such as Real-Time PCR can effectively affirm or deny reference methods.

## Conclusion

Overall, early diagnosis of *Fasciola* species infection is provided by serological methods, but circulating antibodies may remain in the blood for several months after successful treatment. Therefore, serology does not always measure the current infection but only the exposure to the parasite. Our findings provides new insight into the performance of some existing diagnostic tests for the diagnosis of *F. hepatica* infections in the human population. Real-time PCR technique can be a valuable assay in monitoring and understanding the changing epidemiology of *F. hepatica* as well as evaluating population health programs knowing its disadvantages and being able to regulate it. The PCR assay is a comparable test that could be used throughout the year with acceptable sensitivity and specificity. The control of fascioliasis is a global challenge, hence qualitative and quantitative evaluation of existing diagnostic tests as well as development of better field tests are needed.

### Supplementary Information

Supplementary Figure 1.

## Data Availability

All data generated or analyzed during this study are included in this published article.
