# Supplementary material for: Comparative evaluation of real-time PCR and ELISA for the detection of human fascioliasis
Source: Sci Rep. 2024 Feb 16;14:3865. doi: 10.1038/s41598-024-54602-y (PMC10873325; doi:10.1038/s41598-024-54602-y)
Supplement: Supplementary file 1 — Supplementary Figure 1. [file 41598_2024_54602_MOESM1_ESM.doc]

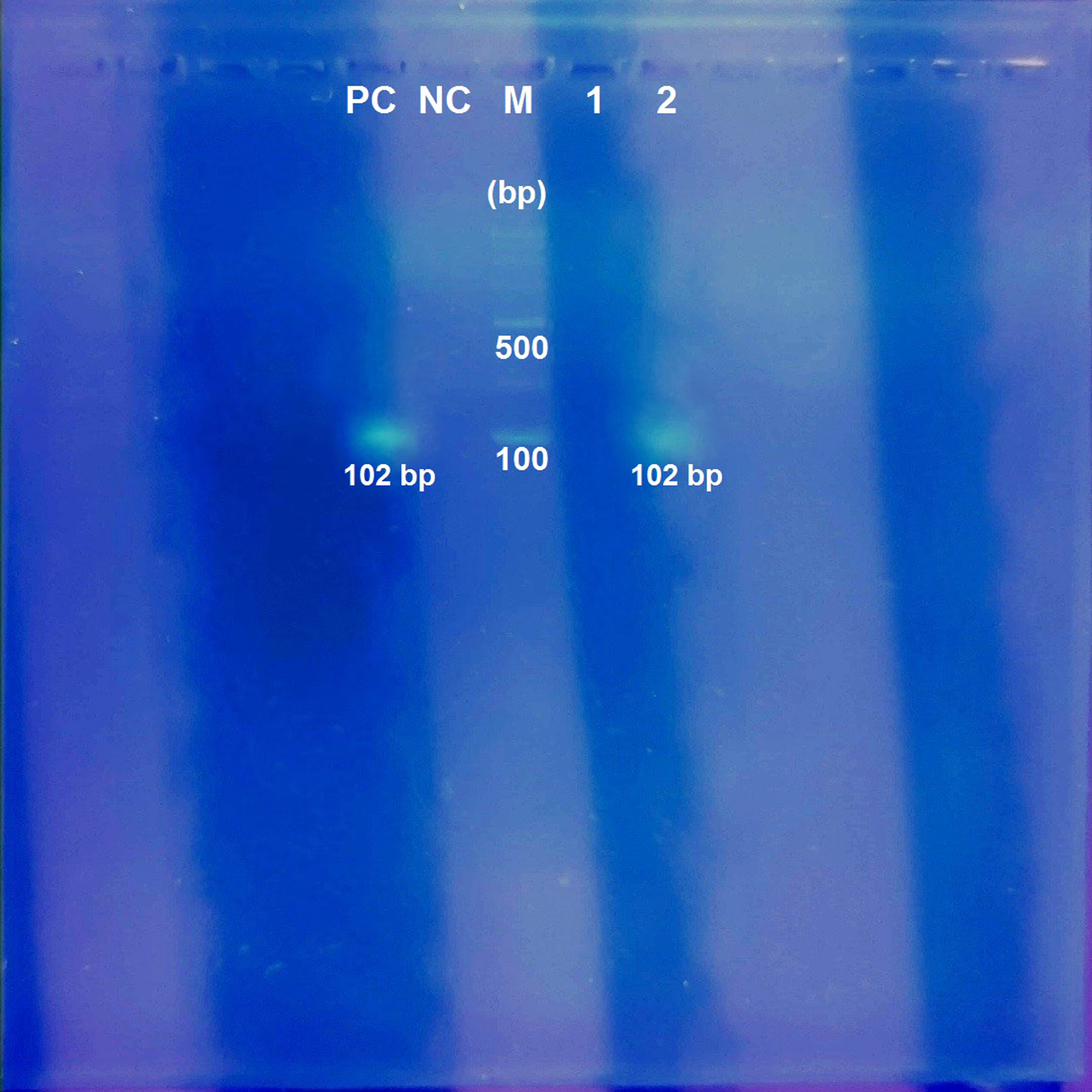


**Additional file 1**

Supplementary Fig. 1. Full-length gel images of PCR assay.

Analysis of PCR products of *Fasciola* species by electrophoresis on 1.5% agarose gels. The findings of the study reveals that the infected sera have *Fasciola* species DNA. Positive control (PC) with standard DNA, negative control (NC) without DNA, and 100-bp molecular size marker (M). Serum sample without *Fasciola* species infection (Lane 1), and the infected sera sample had *Fasciola* DNA amplification of 102-bp fragments (Lane 2).
